# Synergistic hyperinflammation in IgA vasculitis complicated by varicella-induced HLH: a case report

**DOI:** 10.3389/fimmu.2025.1675483

**Published:** 2026-01-12

**Authors:** Xiaoli He, Yu Zhou, Lina Qiao, Deyuan Li, Zhongqiang Liu, Guoyan Lu

**Affiliations:** 1 Department of Pediatrics, West China Second University Hospital, Sichuan University, Chengdu, China; 2 Department of Pediatrics, West China Second University Hospital (WCSUH)-Tianfu·Sichuan Provincial Children’s Hospital, Meishan, Sichuan, China; 3 Key Laboratory of Birth Defects and Related Diseases of Women and Children (Sichuan University), Ministry of Education, Chengdu, China; 4 National Health Commission (NHC) Key Laboratory of Chronobiology, Sichuan University, Chengdu, China

**Keywords:** disseminated intravascular coagulation(DIC), hemophagocytic lymphohistiocytosis(HLH), IgA vasculitis, lymphocytopenia, plasma exchange(PE), varicella zoster virus (VZV)

## Abstract

Varicella-zoster virus (VZV)-induced hemophagocytic lymphohistiocytosis (HLH) is a rare but fatal complication, particularly in immunocompromised hosts. We present an 11-year-old boy with IgA vasculitis who developed severe VZV-HLH complicated by disseminated intravascular coagulation (DIC), acute liver failure and persistent lymphocytopenia. A multimodal therapeutic approach that combining high-dose acyclovir, intravenous immunoglobulin (IVIG), therapeutic plasma exchange (TPE), reduced-dose etoposide (75 mg/m²), and dexamethasone achieved rapid disease remission. This case demonstrates synergistic risk of HLH when VZV infection overlaps with IgA vasculitis, likely via compounded immune dysregulation. This case suggests that when varicella zoster virus infection overlaps with IgA vasculitis, it may synergistically increase the risk of HLH through aggravated immune dysregulation. In infection-triggered HLH, step-wise immunomodulatory therapy has a key role. What’s more, lymphocytopenia may be used as a biomarker to assess disease severity and recovery, and this finding emphasizes the need for long-term immune monitoring. This case provides valuable insights into the pathogenesis and management of VZV related HLH in rheumatic and immune diseases.

## Introduction

Varicella zoster virus (VZV) is a highly contagious double-stranded DNA alpha herpesvirus and it’s the pathogenic agent of varicella (chickenpox) and herpes zoster (shingles) (1). Primary infection typically occurs through inhalation of aerosolized droplets or direct contact with vesicular fluid, following which the virus establishes lifelong latency in sensory ganglia (2).VZV can reactive when body immune state declines, thus resulting in herpes zoster (2). While primary VZV infection typically causes self-limited varicella in immunocompetent children, immunocompromised hosts may develop lethal complications including hepatitis and encephalitis (3). The control of VZV infection relies heavily on the host’s cell-mediated immunity, particularly the recognition and clearance of infected cells by viral-specific CD8+ cytotoxic T lymphocytes, which depend on the presentation of viral antigens via MHC class I molecules (4). Notably, VZV can evade immune surveillance by downregulating MHC class I molecules (5), creating a permissive environment for hyperinflammation. This is particularly relevant to HLH pathogenesis, where impaired viral clearance and excessive cytokine production form a vicious cycle (6). Hemophagocytic lymphohistiocytosis (HLH) is a life-threatening disease caused by dysregulated activation of macrophages, natural killer (NK) cells, and cytotoxic CD8+ T cells, thus resulting in hypercytokinemia, cytopenia, coagulopathy and multiple organ dysfunction (6, 7). Mortality of HLH is high, ranging from 8 to 22% in pediatric, and about 40% in adult (8, 9). HLH can be divided into primary HLH and secondary HLH (7, 8). While primary HLH is caused by genetic mutations that directly impair the cytolytic function of NK cells and CD8+ T lymphocytes, several factors could drive the development of secondary HLH, such as infection, malignancy, drug and primary autoimmune disease (7). The most common and prototypical genetic defects affect the perforin-mediated cytotoxic pathway, including mutations in genes such as *PRF1*, *UNC13D*, *STXBP2*, *STX11*, *SH2D1A*, etc (10). These defects prevent immune cells from efficiently eliminating infected or antigen-presenting cells, leading to uncontrolled immune activation and a rampant cytokine storm, which are the hallmarks of HLH. Among infection-associated HLH cases, EBV is the leading cause, while VZV accounts for only 2.2% of the study population. Mortality of HLH induced by rheumatic diseases is high, ranging from 10-50% (11). The concomitant presence of IgA vasculitis, a condition characterized by aberrant IgA1 glycosylation (12), may further exacerbate immune dysregulation, though direct evidence remains scarce.

Hemophagocytic lymphohistiocytosis (HLH) triggered by varicella-zoster virus (VZV) is uncommon, and its co-occurrence with IgA vasculitis is exceedingly rare. This report describes the case of an 11-year-old boy who developed VZV-associated HLH in the context of active IgA vasculitis. HLH-94 chemoimmunotherapy for primary or non infectious HLH, including dexamethasone and etoposide, has the core goal of inhibiting the overactivated immune system (13). However, when infection and immune disorders intertwine, the potent immune suppression of traditional chemotherapy regimen may exacerbate the infection risk. We aim to illustrate the therapeutic dilemmas posed by this convergence of infection, immune dysregulation, and hyperinflammation. Furthermore, this case serves to highlight the critical need for treatment strategies that can effectively control the life-threatening hyperimmune response without exacerbating the underlying infection.

## Case description

An 11-year-old boy, who was clinically diagnosed with IgA vasculitis one month prior according to the EULAR/PRINTO/PRES criteria, presented to our nephrology department. His initial presentation included the symmetric palpable purpura on the lower extremities, arthritis, and abdominal pain, responding well to corticosteroid therapy. As there was no evidence of kidney involvement at disease onset (normal urinalysis), a renal biopsy was not performed. The patient was discharged with instructions to take a full course of oral prednisone, but he discontinued the medication after 8 days due to nonadherence. He was readmitted with the complaint of new vesicular rash, which disseminated within 48 hours of his second hospitalization, necessitating transfer.

Further history revealed recent exposure to a shingles patient in the same ward. On admission, vital signs were stable. Elevated white blood cell (18.33 x 10(9)/L, normal range <11.9 x 10(9)/L), neutrophils (15.79 x 10(9)/L, normal range <7.0 x 10(9)/L) and normal C-reactive protein was found on admission. Sonoclot coagulation analysis indicated increased D-Dimer (1.44mg/L, normal range <0.55mg/L FEU) and prolonged PT (26.4 sec, normal range <13.2 sec). Urine test showed proteinuria (urine protein2+) and hematuria (RBC 1+). The ALT was 252U/L (normal range <49U/L), AST level was 244.7 U/L (normal range <40U/L), renal function and blood lipids kept in normal range. Other laboratory tests showed that serum creatinine, urea, and complement levels (C3, C4), immunoglobulin were within the normal range. Antineutrophil cytoplasmic antibodies, autoantibodies, anti-cardiolipin antibodies were all negative. Varicella zoster virus nucleic acid was positive. The detailed laboratory parameters during hospitalization are presented in **Table 1**. Initial treatment with IV acyclovir and IVIG was administered. However, the child developed persistent high fever, hemorrhagic rash progression and oral mucosa bleeding during hospitalization. Due to clinical deterioration, he was transferred to the pediatric intensive care unit on hospital day 3. The detailed laboratory parameters during hospitalization are presented in Table 1.

**Table 1:** Major laboratory results during hospitalization.

Parameters	Day1	Day3	Day6	Day8	Day9	Day11	Day17	Day24	Day32	Day36
WBC,/ul	18.33	7.66	1.79	1.7	1.01	0.81	0.660	18.25	5.1	5.86
Hb, g/dl	141	146	102	94	88	70	77	120	109	113
Paltelets,/ul	109	3	13	45	20	36	71	207	339	342
Neutrophil,/ul	15.79	4.52	1.16	1.09	0.48	0.63	0.18	11.68	4.08	4.57
Lymphocyte,/ul	1.94	2.22	0.55	0.48	0.22	0.19	0.38	2.01	0.64	0.89
CRP,mg/dL	1.26	7.76	3.02	2.87	4.62	2.71	0.2	0.37	0.2	0.2
PCT, ng/ml	–	1.18	0.68	0.87	0.52	–	–	0.33	0.06	–
AST, U/L	244.7	1918.4	407	250.7	224.3	–	71.2	47.4	–	–
ALT, U/L	252	1530.2	405	144.8	101.5	–	86.9	74.6	–	–
LDH, U/L	1378.3	>5000	4221	2858.4	2668.5	–	631.9	561	–	–
GGT, U/L	39.4	313.1	294.5	169.7	128.7	–	187.8	164.8	–	–
TG, mmol/L	0.92	–	1.81	–	1.89	–	1.81	–	–	–
Ferritin, ng/ml	–	–	11885.2	–	8339.52	6592.49	2721.31	1078.7	352	–
CMV-DNA copies, x10^3^	–	–	1.93	–	–	1.27	–	<1	–	<1
DDI, mg/L	1.44	>40	>40	>40	>40	>40	39.92	29.09	10.83	6.82
PT, S	26.4	59.9	12.3	12	11	11	10.8	10.6	10.7	9.9
INR	1.11	2.33	1.06	1.03	0.93	0.93	0.91	0.9	0.91	0.83
FDP, ug/ml	4.79	>656	103.78	107.04	97.96	68.61	68.49	30.1	13.48	8.86
APTT, s	26.4	41.3	27.9	27.9	24.2	23.3	20.3	24.4	20.9	21.9
Fg, g/L	2.73	1.01	0.87	0.84	1.59	1.49	2.62	2.28	1.65	8.86
TT, s	15.3	25.1	42.8	54.1	24.5	19.7	16.3	22.5	19.7	20.5
CD3+, x10^9^	1.52	–	0.58	–	–	0.1	–	–	–	–
CD3+CD4+, x10^9^	0.6	–	0.08	–	–	0.04	–	–	–	–
CD3+CD8+, x10^9^	0.77	–	0.49	–	–	0.06	–	–	–	–
CD19+, x10^9^	0.41	–	0.04	–	–	0.02	–	–	–	–
CD3-CD16 + 56+, x10^9^	0.12	–	0.03	–	–	<0.01	–	–	–	–

CRP, C-reactive protein; PCT, procalcitonin; AST, aspartate aminotransferase; ALT, alanine aminotransferase; LDH, lactate dehydrogenase; GGT, γ-glutamyl transpeptidase; TG, triglycerides; DDI, d-dimer; PT, prothrombin time; INR, international nomalized ratio; FDP, fibrinogen degradation products; APTT, activated partial prothrombin time; Fg, fibrinogen; TT, thrombin time.

**Figure 1:**
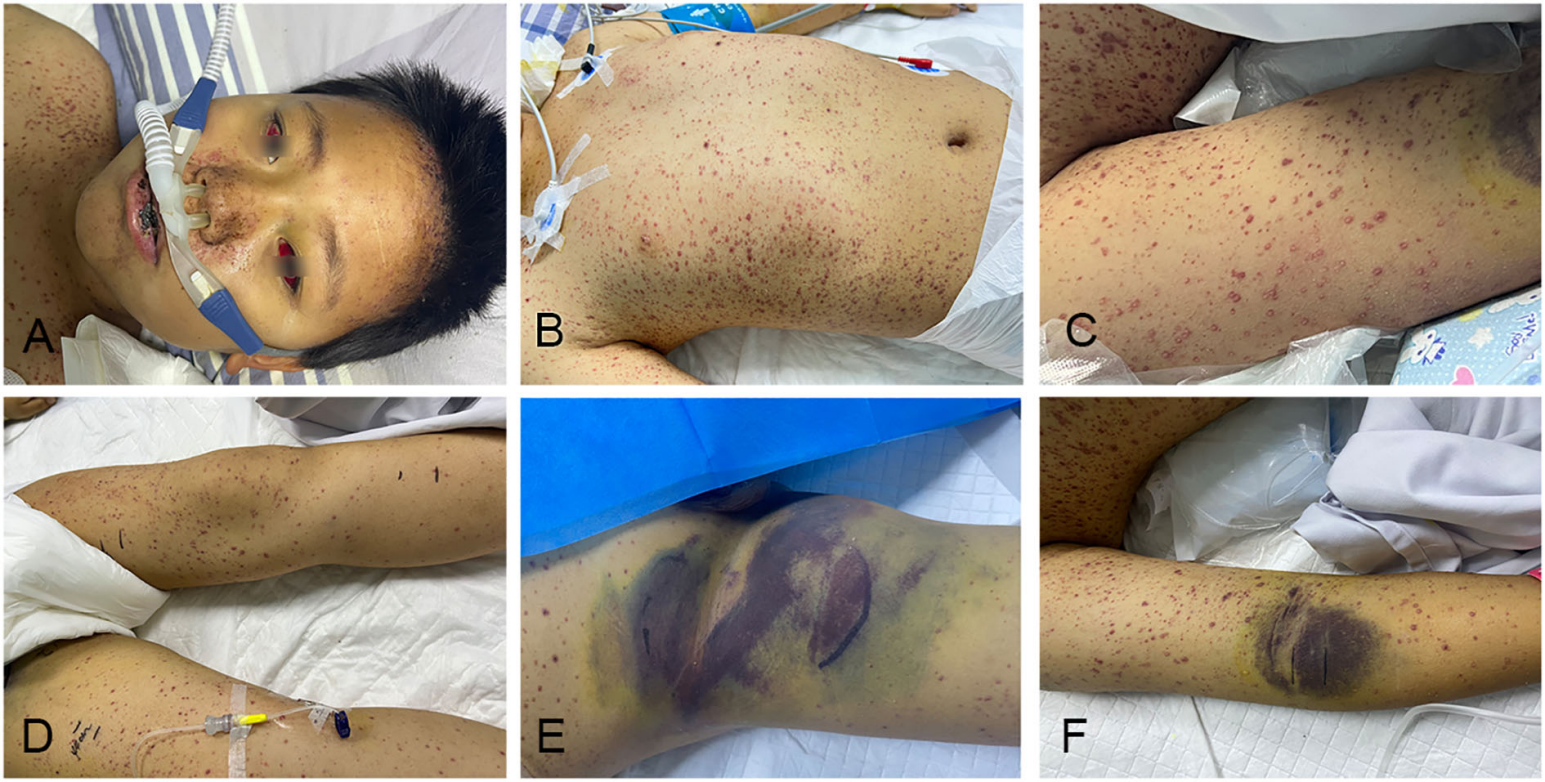
Skin manifestations. **(A–D)** the patient had rashes on the face **(A)**, trunk **(B)**, arms **(C)** and legs **(D)**, bulbar conjunctival hemorrhage was also shown in **(A)**; **(E, F)** ecchymosis and hematoma at groin puncture site **(E)** and right cubital fossa puncture site **(F)**.

Upon PICU admission, physical examination showed tachycardia (154 bpm), tachypnea (33 breaths/min), high fever (39.7°C), warm extremities with normal capillary refill (2s), extensive hemorrhagic-vesicular rash with ulceration (predominantly on chest/back), accompanied by bulbar conjunctival hemorrhage, persistent oral mucosal bleeding, ecchymosis at puncture sites (**Figure 1**). Physical examination of the abdomen revealed splenomegaly. Initial interventions included high-flow nasal cannula oxygen for respiratory support, along with empiric antimicrobial therapy using imipenem, vancomycin, and micafungin for sepsis prophylaxis. Targeted therapies consisted of intravenous methylprednisolone (0.5 mg/kg/day), high-dose acyclovir (800 mg every 6 hours, approximately 20 mg/kg per dose for a 40 kg child), and intravenous immunoglobulin (20 g on days 1-2, 42.5 g on day 3, 20 g on day 4, and 7.5 g on days 7, 8, 10, and 11; cumulative dose of 2.7 g/kg over the hospitalization period.

On the first day of ICU admission (hospital day 3), laboratory investigations revealed critical hematologic and biochemical disturbances, showing severe thrombocytopenia (3×10^9^/L, normal range>125×10^9^/L), consumptive coagulopathy (D-dimer >40 mg/l FEU, normal range <0.55 mg/l FEU; fibrinogen 1.01 g/L, normal range 0.2~0.4 g/L; PT 25.1s, normal range <13.2S; INR 2.33, normal range <1.5), and acute hepatocellular injury (ALT 1530.2 U/L, AST 1918.4 U/L, approximately 6-fold transaminase elevation from admission). The patient’s cytokine levels are significantly elevated (IL-2R 6176.7U/ml, normal range <710 U/ml; IL-8 51.89pg/ml, normal range <21.4 pg/ml; IL-10 706.33 pg/ml, normal range <5.9 pg/ml; IFN-γ 441.32 pg/ml, normal range <17.3 pg/ml). Laboratory investigation on admission showed normal levels of serum immunoglobulins (IgG, IgA, IgM). Lymphocyte subset analysis on the second day of admission was in normal range. Virologic studies demonstrated significant CMV viremia (1.93×10^9^ copies/L) alongside persistent VZV infection, while comprehensive microbiologic workup excluded other pathogens including EBV, hepatitis viruses, SARS-CoV-2, and bacterial/fungal organisms. The blood metagenomic next-generation sequencing (mNGS) later confirmed active infection with CMV and VZV. The patient was diagnosed with severe varicella complicated by sepsis, disseminated intravascular coagulation, acute liver failure and IgA vasculitis-associated nephritis (hematuria-proteinuria subtype).

**Figure 2:**
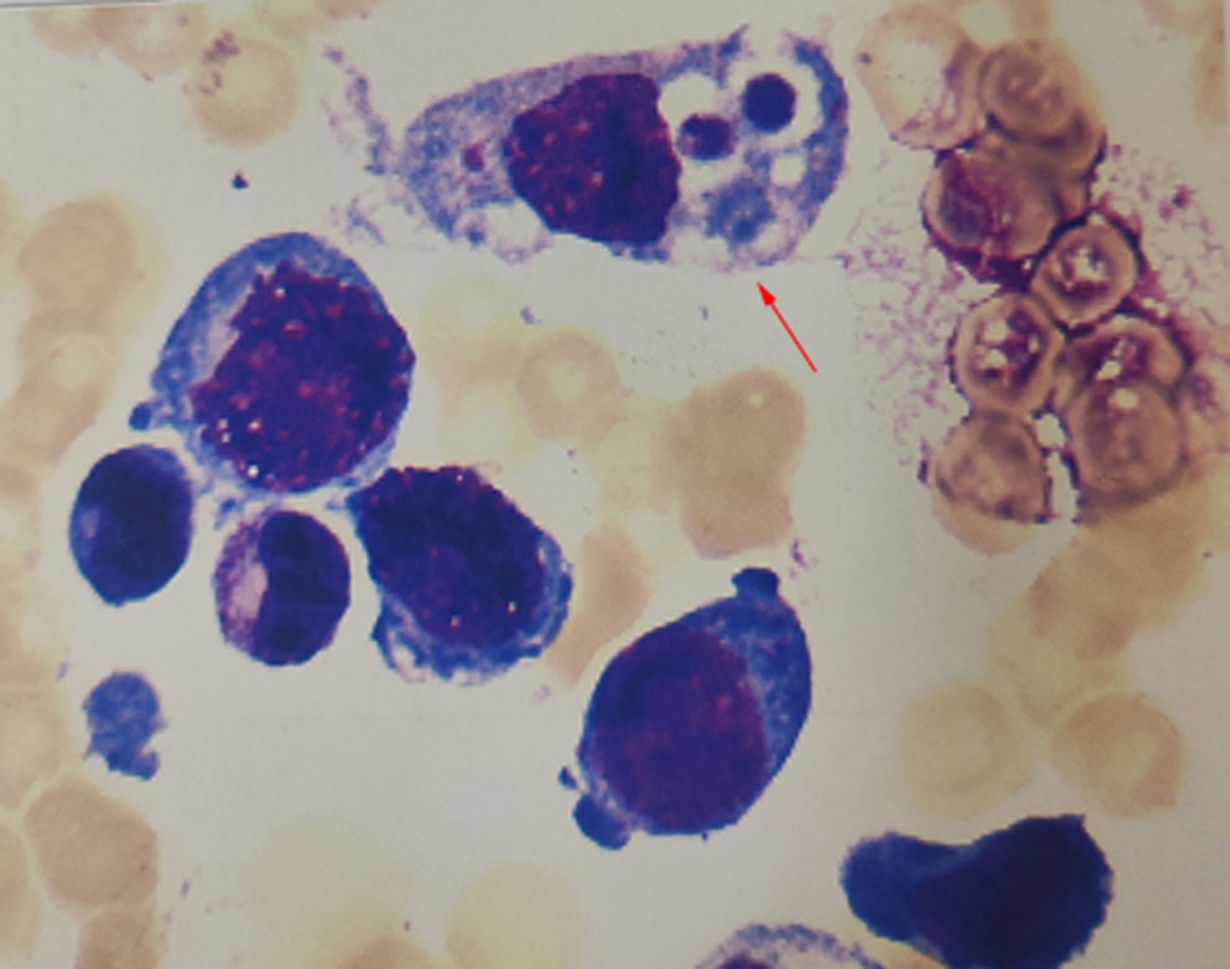
Bone marrow smear of the patient. Red arrow indicated hemophagocytic cells.

Initial management comprised ganciclovir for CMV coinfection, ongoing high-dose acyclovir (800 mg q6h), continuous intravenous immunoglobulin (IVIG), aggressive blood product support (plasma/platelet transfusions) and hepatoprotective therapy. Despite transient improvement in liver enzymes by day 6, the patient developed persistent fever with progressive pancytopenia (lowest WBC 0.81×10^9^/L, absolute neutrophil count 0.63×10^9^/L, hemoglobin 70 g/L), extreme hyperferritinemia (11,885.2 ng/mL, normal range <322 ng/mL, firstly obtained during hospitalization), and hypofibrinogenemia (nadir 0.84 g/L). Lymphocyte subset analysis revealed a severe depletion of B, NK, and T cells. Specifically, the results demonstrated critically low counts of B lymphocytes (0.02 x 10^9^/L; normal >0.20), NK lymphocytes (<0.01 x 10^9^/L; normal >0.07), and total T lymphocytes (0.1 x 10^9^/L; normal >0.80) Notably, helper T cells were markedly reduced (0.04 x 10^9^/L; normal >0.40), whereas suppressor T cell counts remained within the normal range (0.06 x 10^9^/L; normal >0.40). The lowest lymphocyte count about 0.22×10^9^/L(normal range 1.5~4.6×10^9^/L). These findings, coupled with the bone marrow demonstration of hemophagocytosis (**Figure 2**), fulfilled six of the eight HLH-2004 diagnostic criteria (fever, splenomegaly, cytopenias, hypofibrinogenemia, hemophagocytosis in bone marrow, elevated ferritin). Immediate escalation to HLH-directed therapy included three consecutive plasma exchange sessions (days 5-7) and reduced dexamethasone (5 mg/m²/day, administered as 2.5 mg/m² every 12 hours, on day 6~25), while maintaining antiviral coverage and supportive care.

**Figure 3:**
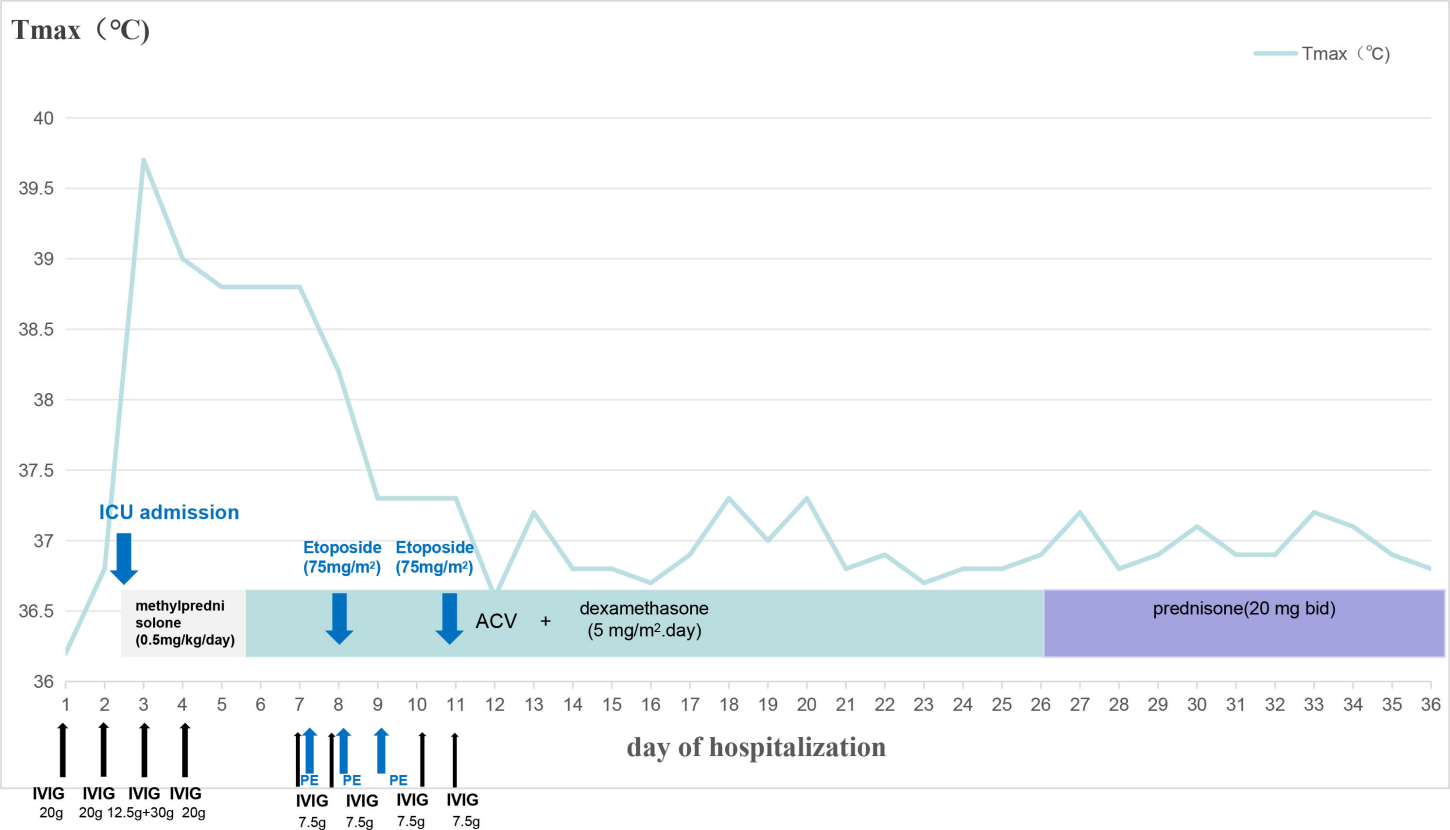
Time course treatments for VZV-induced HLH.

Continuing from the previous management, the patient’s persistent fever and pancytopenia improved since the initiation of reduced-dose etoposide chemotherapy (75 mg/m² ×2 doses) alongside continued dexamethasone. Prophylactic sulfamethoxazole-trimethoprim was administered for Pneumocystis jirovecii prevention. This therapeutic regimen achieved rapid clinical response, with normalization of body temperature within 48 hours (**Figure 3**) and progressive improvement in hematologic parameters. Following approximately three weeks of intravenous dexamethasone, the steroid regimen was transitioned to an oral taper with prednisone (20 mg twice daily) on hospital day 26 as part of the planned steroid taper. Following a 33-day intensive care course, the patient was successfully discharged with complete resolution of both proteinuria and hematuria, demonstrating full renal recovery. Final laboratory evaluation showed sustained remission of both the viral infections and HLH, with platelet count recovering to 339×10^9^/L and ferritin declining to 352 ng/mL at discharge.

During the following 2 months, the boy was followed up regularly. While liver and renal function parameters and ferritin levels were maintained within normal limits, persistent lymphocytopenia was detected post-discharge. And the lymphocyte level did not return to normal range until two months after discharge. The parents of the boy felt satisfied with the treatment.

## Discussion

Hemophagocytic lymphohistiocytosis (HLH) represents a life-threatening hyperinflammatory syndrome with diverse triggers. While both rheumatic diseases and viral infections are well-established independent causes of HLH, the specific scenarios of IgA vasculitis (IgAV) or varicella-zoster virus (VZV) infection alone rarely induce this complication. This observation raises important questions about potential synergistic mechanisms when these conditions coexist.

The pathophysiology of HLH typically involves excessive cytokine release (particularly IFN-γ, IL-6, and IL-18) coupled with impaired NK/CD8+ T cell cytotoxicity (7). In rheumatologic contexts, systemic juvenile idiopathic arthritis (sJIA) demonstrates the strongest association, with approximately 10% of patients developing secondary macrophage activation syndrome (MAS), where disease severity correlates strikingly with IL-18 levels (14). Other connective tissue diseases including systemic lupus erythematosus, dermatomyositis, and Kawasaki disease also feature established HLH risk (15, 16). Similarly, viral pathogens, especially Epstein-Barr virus (EBV) and cytomegalovirus (CMV), frequently trigger HLH, particularly in immunocompromised hosts (8, 17, 18).

Notably, isolated IgAV (Henoch-Schönlein purpura) seldom progresses to HLH, with only sporadic case reports documenting this progression (19). The proposed mechanisms in these rare instances include IgA-mediated macrophage activation through immune complex deposition and secondary infection-triggered cytokine storms. Parallel observations exist for VZV, which ranks among the least common viral HLH triggers despite its prevalence in pediatric populations (19–22). The clinical convergence of IgAV and VZV infection presents a scientifically compelling scenario. Documented cases including a 5-year-old girl with HSP developing VZV-induced HLH (19) and our current pediatric case suggest this combination may overcome the individual thresholds for HLH development. We hypothesize five potential interactive mechanisms. Firstly, the VZV infection may amplify IgA-mediated vascular inflammation through molecular mimicry or epitope spreading. Secondly, pre-existing endothelial damage from IgAV could facilitate viral dissemination and immune disorders. Thirdly, the presence of a concomitant CMV infection, as evidenced by the high viral load in our patient, must be considered as a significant co-factor. CMV is a potent driver of T/NK-cell activation and exhaustion, and its synergy with VZV could have delivered a compounded antigenic load that overwhelmed immune regulatory capacity, thereby triggering and sustaining the hyperinflammatory state of HLH (23). Fourthly, the concurrent stimulation from VZV/CMV infection and IgAV triggers may overwhelm regulatory pathways. Finally, a preceding or concurrent course of corticosteroid therapy for the vasculitis would have induced iatrogenic immunosuppression, which was considered as the key factor for disease severity (24). This significantly impairs T-cell and NK-cell mediated viral clearance, creating a permissive environment for uncontrolled VZV replication, which in turn acts as a potent trigger for the cytokine storm characteristic of HLH. This multifactorial model challenges the traditional “single-trigger” paradigm and underscores the need for therapies addressing both viral and autoimmune components. Such complexity necessitates a novel therapeutic strategy that simultaneously target viral clearance, endothelial protection, and controlled immunomodulation, which remains a challenge for the conventional HLH protocols.

It is increasingly recognized that HLH can arise not only in classic “primary” forms or in overt immunodeficiency but also on a spectrum of immune dysregulation, including in patients with underlying inborn errors of immunity (IEI) or autoimmune/autoinflammatory diseases like IgAV itself (25, 26). Given the presentation of hemophagocytic lymphohistiocytosis (HLH) following IgA vasculitis, an underlying inborn error of immunity (IEI) was considered in the differential diagnosis. However, several factors in our patient argue against this. Firstly, the child had normal growth and development, with no history of recurrent or severe infections prior to this acute episode. Secondly, he was previously healthy, with normal cellular and humoral immunity parameters at the onset of his illness. The subsequent decline in lymphocyte subsets paralleled the progression of HLH, a pattern consistent with immune cell consumption due to hyperinflammation rather than a primary immunodeficiency. Regarding the poor VZV control of the patient, we believe it can be adequately explained by the synergistic effect of iatrogenic immunosuppression and the immune-dysregulated state of active IgA vasculitis. The corticosteroid therapy administered for the vasculitis is a well-established risk factor for impairing cell-mediated immunity, which is crucial for controlling VZV replication. This, combined with the profound inflammatory environment of HLH itself—which leads to T-cell exhaustion and impaired cytotoxic function—likely created a ‘perfect storm’ that permitted viral dissemination, rather than pointing to an underlying genetic defect in antiviral immunity. Thus, the HLH is most accurately classified as secondary to IgA vasculitis rather than to an inborn error of immunity, despite the lack of comprehensive genetic testing.

While the HLH-94 regimen (dexamethasone/etoposide) remains foundational for primary HLH by suppressing hyperinflammation (7, 13), its unmodified application in infection-associated cases risks exacerbating pathogen dissemination. Notably, VZV-triggered HLH has achieved remission without chemotherapy in selected patients (e.g., Jun et al.’s nephrotic syndrome case (20) and Gökçe Gür’s IgAV patient (19), via combined high-dose acyclovir, IVIG, plasma exchange and corticosteroids. These successesful cases highlight three therapeutic axes, namely viral control (PE/antiviral treatment reduce viral load), cytokine modulation (PE removes inflammatory mediators) and immune balancing (IVIG/steroids mitigate the immune dysregulation).

However, this approach may prove insufficient for refractory cases or those with high-risk features, which was exemplified by our patient. In conjunction with the prior regimen, we applied a step-up protocol combining reduced-dose etoposide (75 mg/m² per dose, administered twice in the first week only), attenuated corticosteroid use to limit immunosuppression, and continuous IVIG to reinforce immune function. This approach led to rapid clinical and laboratory improvement, mirroring outcomes in Zhang et al.’s severe VZV-HLH case with acute liver failure (22). These findings highlight a critical paradigm, while mild-to-moderate VZV-associated HLH may respond to chemotherapy-free regimens, early escalation to reduced-dose etoposide is still warranted in refractory cases, especially for patients with risk factors, such as cytopenia, organ dysfunction, high viral load persistence, persistent lymphocytopenia, or inadequate response within 72 hours. This risk-adapted framework optimizes the balance between immunosuppression and infection control, offering a tailored therapeutic pathway for infection-triggered HLH.

The patient in our case showed prolonged lymphocytopenia, persisting for two months post-discharge, accompanied by neutropenia. This illustrates a clinically significant yet underreported phenomenon in varicella-associated hemophagocytic lymphohistiocytosis (HLH). This protracted immunosuppressive state carries critical clinical implications, bridging our understanding of VZV associated hemophagocytic lymphohistiocytosis (VZV-HLH) with that of other severe viral infections. This pattern aligns with observations from other severe infectious illness. In COVID-19, lymphocytopenia correlate with higher risk for developing severe COVID-19 (27) and lymphocytopenia was also associated with greater mortality (28). Sepsis studies by Tang et al. identified CD8+ T-cell depletion as a reliable marker for disease progression (29). The delayed lymphocyte recovery in our case can give us some suggestions. VZV-specific immune exhaustion and ongoing subclinical HLH activity may cause bone marrow suppression. Firstly, it serves as a prognostic marker, probably indicating higher risks of secondary infections or disease relapse. What’s more, it also indicates that conventional IVIG may not be effective for immune reconstitution, and it’s urgent to extend immune monitoring and prolonged antiviral prophylaxis. That’s to say, lymphocytopenia is not merely a laboratory abnormality, but also a clinically significant phenomenon that warrants systematic monitoring, which may guide the treatment of VZV-HLH.

This report enriches the documented cases of IgA vasculitis complicated by VZV-associated HLH (VZV-HLH) in pediatric populations. Our findings provide several critical insights. Firstly, early therapeutic plasma exchange (TPE) prior to etoposide chemotherapy safely achieves cytokine reduction, addressing a key knowledge gap. This supports the “two-hit hypothesis” of HLH pathogenesis (immune activation combined with impaired cytotoxicity) and offers a human model for optimizing the timing of immunomodulatory therapy post-viral infection. In addition, the case demonstrates a balanced therapeutic strategy for immunosuppressed patients, reconciling the dual demands of viral infection control (high-dose acyclovir) and immune suppression (dexamethasone/etoposide). Finally, persistent lymphocytopenia may indicate severity of the condition, warranting close monitoring of immune recovery.

## Conclusion

IgA Vasculitis Complicated by Varicella-Induced HLH represents a complex and life-threatening hyperinflammatory syndrome with unique pathophysiological and therapeutic challenges. For mild cases, first-line non-chemotherapy approaches, such as high-dose acyclovir, IVIG and plasma exchange, may suffice particularly for patients at an early stage of the disease episode. For refractory cases, a reduced-dose etoposide protocol may be urgent to balance the underlying immunosuppression and infection management. Persistent lymphocytopenia may serve as an indicator for disease severity, warranting close monitoring of immune recovery. The underlying mechanisms contributing to VZV-triggered HLH development, along with the clinical relevance of lymphopenia as a prognostic marker require further investigation in larger cohort studies.

## Data Availability

The original contributions presented in the study are included in the article/supplementary material. Further inquiries can be directed to the corresponding author.
